# Trajectories of Social Activity Engagement and Physical and Cognitive Function During the Last Years of Life

**DOI:** 10.1111/jgs.70410

**Published:** 2026-04-01

**Authors:** Sara M. Moorman, Alyssa W. Goldman

**Affiliations:** Department of Sociology, Boston College, Chestnut Hill, Massachusetts, USA

**Keywords:** dementia, end-of-life, functional health, growth curve modeling, social support

## Abstract

**Background:**

Remaining socially active may be especially critical for quality of life as death approaches, as it affords access to necessary support, health advice, a sense of autonomy, and personal resources (e.g., coping, resilience) that shape end-of-life experiences. We aimed to examine (a) how older adults’ engagement in social activities changes during the years before death, and (b) whether trajectories of engagement in these activities differ depending on physical and cognitive function.

**Participants and Setting:**

We analyzed 22,993 annual observations from 4667 participants in the National Health and Aging Trends Study (NHATS), a U.S. nationally-representative, longitudinal study of Medicare recipients aged 65 and older who died between 2012 and 2024.

**Methods:**

We used logistic growth curve models to test how participation in each of 5 activities—visiting with family or friends, religious service attendance, organized group activities, going out for enjoyment, and volunteering—changed over the 13 years prior to death. Key predictors included physical and cognitive function.

**Results:**

Findings indicated that older adults’ participation in all 5 social activities declined during this final period of the life course, with considerable variation across activities. There was strong evidence that physical and cognitive function affected initial levels of participation, but minimal evidence that physical or cognitive function affected trajectories of decline.

**Conclusions:**

Community-based interventions to improve physical accessibility and dementia awareness may be important avenues for supporting social activity engagement among people in the last stage of life, particularly among older adults living independently in their communities.

Engagement in social activities—leisure or recreational activities that involve one or more other persons—is essential to well-being across the life course, including in later life [1–3]. Older adults who participate in social activities may have greater access to social support, including opportunities to form new social ties, exchange advice and influence, maintain diverse social roles, and cultivate a sense of meaning and purpose in life [4–7]. These factors are associated with myriad indicators of physical, mental, and cognitive well-being, including mortality risk [1, 8, 9].

Examining changes in social activities in later life is critical to assessing the potential risks associated with instability in or loss of key social resources in the face of life course transitions that can disrupt patterns of social activity participation (e.g., bereavement, retirement) [10–13]. Studies have yielded varied findings, with some evidence that social activity declines with age, but other evidence suggests that changes are more nuanced, including stability or even growth in social activity and other forms of social connectedness for some older adults [14–17].

Most research has focused on patterns of change in various aspects of social life across decades of older adulthood broadly, with less specific attention to change during the final years of life, which can exhibit great age variation [18]. The nature of social activity engagement may change in the final years of life as individuals choose to spend less time forming new relationships and more time engaging with intimate, long-standing social ties [16, 19]. Yet remaining socially active, even in a changed form, may be especially critical for quality of life as death approaches, as it affords access to necessary support, health advice, sense of autonomy, and personal resources (e.g., coping, resilience) that shape end-of-life experiences. Approximately 30%–40% of older adults in the U.S. report experiencing social isolation and/or loneliness during the years prior to death, indicating that some older adults are not able to reap the benefits of social interaction [20, 21].

Although many older adults have chronic illnesses, the extent and duration of physical and cognitive function exhibit considerable heterogeneity at advanced ages, which may contribute to variation in levels of social activity engagement [22, 23]. Impairments to physical and cognitive function pose well-established barriers to participation in social activities [24, 25]. Physical impairments can impede the level of mobility and the sense of safety necessary to participate in certain social activities [26]. Aspects of the built environment that limit opportunities for older persons with physical disabilities to engage socially may further compound both actual and perceived disabilities [27]. Impaired cognitive function can also limit older adults’ social activity participation. Difficulties with working memory, speech, and executive function, for example, can negatively affect individuals’ abilities to understand social roles, form and maintain social relationships, coordinate social activities, and reciprocate social exchanges [28, 29].

Our aims in this paper are twofold. First, we examine how older adults’ participation in social activities changes during the years before death. We consider changes in participation in five different types of social activities: visiting with family or friends, religious service attendance, organized group activities, going out for enjoyment, and volunteering. Second, we examine whether trajectories of participation in these activities in the years before death differ depending on cognitive and, separately, physical function. Impaired physical and cognitive function may be associated with declines in participation due to the inability to physically access or cognitively engage in social opportunities. Alternatively, older adults with health impairments may seek out social activity to maintain a sense of fulfillment or belonging, or as a source of health-related support. Whether older adults maintain levels of social activities as death approaches may depend on whether cognitive and physical function enable or constrain these forms of engagement.

## 1 | Methods

### 1.1 | Data

We analyzed data from the National Health and Aging Trends Study (NHATS), a U.S. nationally representative, longitudinal study of Medicare recipients. Participants were invited to complete annual individual-level interviews by self- or proxy-report. NHATS participants included both community-dwelling older adults and those living in residential care facilities. For participants who lived in long-term care facilities, facility staff supplemented the individual-level interview with a questionnaire concerning characteristics of the facility (e.g., total number of residents).

NHATS began in 2011 with 8245 respondents (response rate: 71%). The study was replenished with new respondents in 2015 and in 2022–2023. Between 2011 and 2024, 5523 participants died. Most decedents (4667, or 85%) had completed at least one self- or proxy-report interview (The remaining decedents had no individual-level data, but rather, only reports from the residential care facilities in which they lived). Because our measures were from individual-level interviews, our sample comprised of 22,993 observations from 4667 decedents. There were 5 (SD = 3) individual-level interviews on average for each decedent, with a range from 1 to 13 (For further detail, see Table S1).

### 1.2 | Dependent Measures

Annually, both self- and proxy-reporters answered 5 yes/no questions about the older adult’s social participation in the past month. Activities included: (1) visit in person with friends or family not living with you, either at your home or theirs; (2) attend religious services; (3) participate in clubs, classes, or other organized activities; (4) go out for enjoyment; and (5) do volunteer work. The item about going out for enjoyment further specified “This includes things like going out to dinner, a movie, to gamble, or to hear music or see a play.” We analyzed the 5 binary items separately.

### 1.3 | Measures of Time

The primary measure of time was years until death, with death represented as 0 for all participants. Therefore, a hypothetical decedent who self-reported in each of the 3 years before death had valid data for years −3, −2, and −1. A hypothetical decedent who self-reported once, 5 years before death, and then had proxy reports until 2 years before death had valid data for years −5, −4, −3, and −2. We also tested a squared term with a negative sign; for example, the squared value corresponding to year −3 was −9.

### 1.4 | Physical Function

Respondents received 6 pairs of questions about the older adult’s physical capacity [30]. First, reporters responded to a question about whether they could perform a task. If the older adult could perform it, they earned two points and moved to the next pair of questions. If the older adult could not perform it, they responded to a question about whether they could perform a less difficult task in the same domain. If the older adult could perform the less difficult task, they earned one point, and if they could not, they earned no points. Therefore, scores ranged from 0 to 12, with higher scores indicating better physical function. The pairs included: walk 6 blocks/3 blocks; walk up 20 stairs/10 stairs; carry 20 pounds/10 pounds; get down on your knees/bend over; put a heavy book or other object on a shelf above your head/reach up over your head; and open a sealed jar using just your hands/grasp or handle small objects. Each item specified “by yourself,” and without aids such as a cane.

### 1.5 | Cognitive Function

Both self- and proxy-reporters responded annually to whether a doctor had ever diagnosed the older adult with dementia or Alzheimer’s disease. Additionally, self-report respondents completed annual tests of orientation (date and US political leaders), memory (immediate and delayed word recall), and executive function (clock draw). Even when proxies completed the individual-level interview, interviewers attempted to complete the 3 cognitive tasks with the older adult. If the older adult was unable, the proxy completed the AD8 Dementia Screening Interview [31]. NHATS combined this information to create a variable classifying participants as “probable,” “possible,” or “no” dementia annually [32]. Those classified as having probable dementia included: (1) anyone with a doctor’s diagnosis, (2) proxy respondents who scored 2 or higher on the AD8, and (3) self-reporters who scored 1.5 SDs or more below average on any two domains (i.e., orientation, memory, and executive function). Those classified as having possible dementia included self-reporters who scored 1.5 SDs or more below average on any one domain. Many participants in the dataset did not progress smoothly from normal cognition to possible dementia to probable dementia; rather, some received a subsequent score of “normal” in a year after having scored “possible.” Therefore, we recoded all “possible” observations as observations of normal cognition (i.e., potential false positives).

### 1.6 | Covariates

Descriptions of covariates are available in Table S2. Time-invariant covariates included year of study entry, year of birth, sex, race and ethnicity, educational attainment, number of children, and logged income. Time-varying covariates included whether the interview was completed by a proxy interviewee, whether the participant had been a caregiver or worked for pay in the past month, whether the participant lived in the community or in a residential care setting, and marital status.

### 1.7 | Analytic Plan

#### 1.7.1 | Statistical Modeling

Analyses were performed using Stata version 18. We used logistic growth curve models to test how participation in each of the 5 activities changed over time. A growth curve model estimates the rate of within-person change in a repeated measure, as well as how that rate of change varies across respondents, in a longitudinal study [33]. This statistical approach is well-suited for the present study because the NHATS consistently measures each of the 5 forms of social participation in its annual survey, allowing us to examine how respondents’ rate of participation changes over the course of the observation period while also adjusting for relevant covariates. First, we modeled a decedent’s outcomes as a function of linear and squared time, controlling only for year of study entry and respondent type (self vs. proxy). The squared term allowed the rate of change to accelerate or decelerate as death approached. Models included a random intercept, a random slope on linear time, and the covariance of the two (Random slopes on squared time were not statistically significant in any model, and so we excluded them).

Second, we estimated models by adding physical and cognitive function and all covariates. We represented both forms of function at both the within-person and between-person levels. The between-person variable was the participant’s average score for up to 5 years before death (accounting for the fact that some participants were enrolled in the study for fewer than 5 years before death). For physical function, this score represented the average physical capacity, ranging from 0 to 12. For cognitive function, this score represented the proportion of years of participation at which the participant had probable dementia. The within-person variable was calculated by substracting the person-level average from the annual value [34]. Finally, we added 8 multiplicative interaction terms to test whether within-person and between-person physical and cognitive function affected the linear or squared rates of change.

Missing data was minimal. At the annual level, 94% of decedents had complete individual-level interviews for all of the years in which they were eligible to participate (see Supplementary Table S3). At the participant level, income was the variable with the greatest missingness, with 104 out of 4667 cases (2%). At the observation level, attendance at religious services was the variable with the greatest missingness, with 73 out of 22,993 (0%). We used listwise deletion.

## 2 | Results

Table 1 contains descriptive statistics for all participants and all observations. Participants were born between 1904 and 1956, with the average participant born in 1931. Over half of the participants (56%) were female, and over half (69%) identified as White non-Hispanic. An additional 22% identified as Black non-Hispanic, 6% identified as Hispanic, and 3% identified as another race or ethnicity. Average physical capacity, across all participants and all years, was 6 (SD = 4) on the 0–12 scale. Participants were classified as having probable dementia in 27% of the 22,993 observations. Visiting family and friends in the past month was the most common form of social participation, occurring in 78% of observations. Visiting was followed in frequency by going out for enjoyment (62% of observations), attending religious services (52% of observations), participating in organized activities (29% of observations), and volunteering (16% of observations).

Tables S4 and S5 show more detailed breakdowns. Table S4 shows descriptive statistics for all time-invariant measures by the year in which the participant entered the study. Participant characteristics were generally stable, except that notably, the proportion of Hispanic decedents increased from 5% in 2011 to 29% in 2023, as a result of changes in NHATS’s recruitment strategy [35].

Table S5 shows descriptive statistics for all time-varying measures by the number of years before the participant’s death, including the five forms of social participation. On a scale from 0 to 12, average physical capacity declined from 9.19 (SD = 3) 13 years before death to 5 (SD = 4) 1 year before death. While only 4% of participants were classified as having probable dementia 13 years before death, 44% were so classified 1 year before death. Accordingly, only 3% of participants had a proxy reporter 13 years before death, while 30% of participants had one in the year before death.

**FIGURE 1 |:**
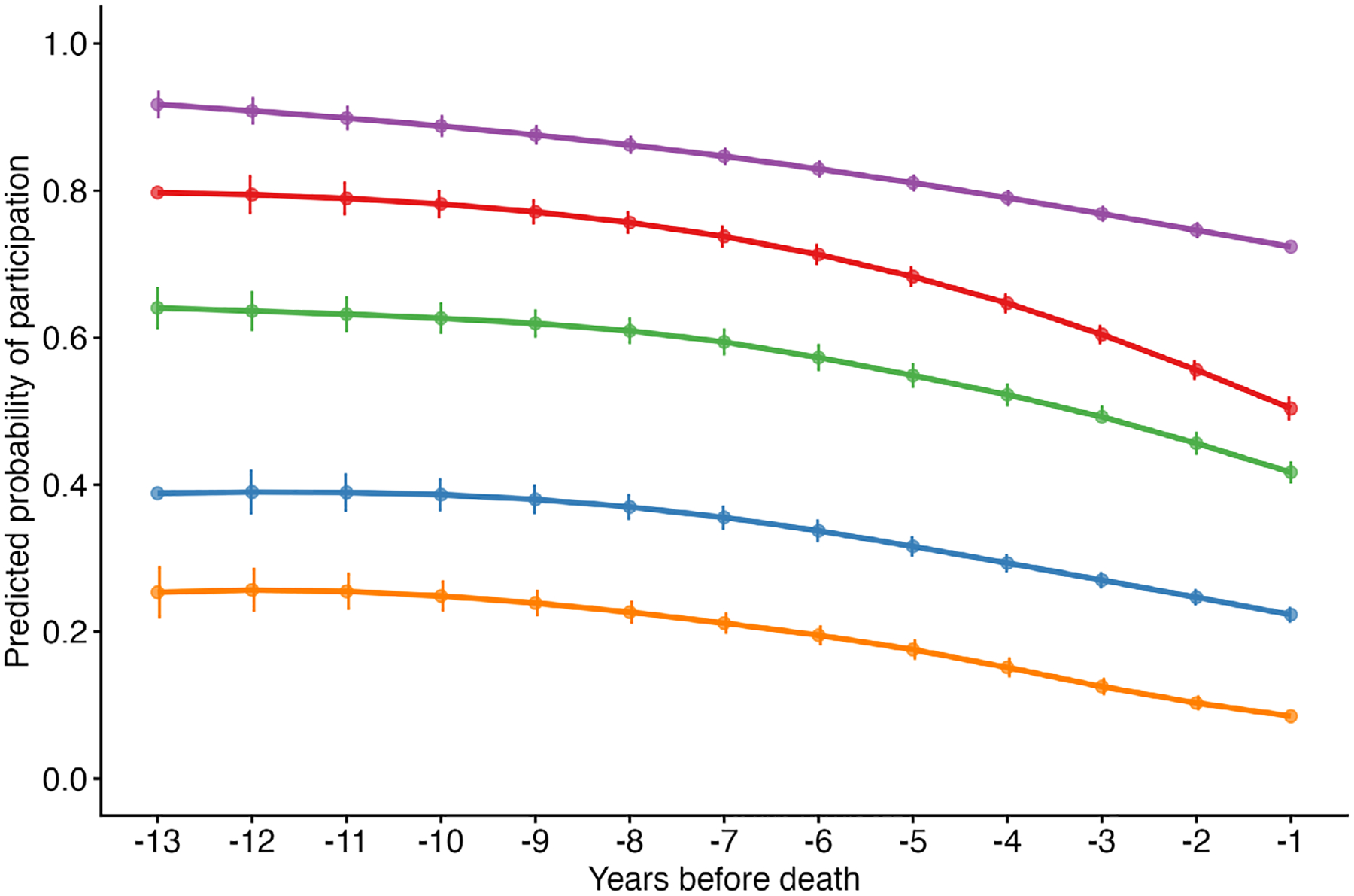
Predicted probabilities of participation in each social activity in the years before death. Purple represents the predicted probability of visiting family and friends in the past month; red represents the predicted probability of going out for enjoyment in the past month; green represents the predicted probability of attending religious services in the past month; blue represents the predicted probability of participating in organized activities in the past month; and orange represents the predicted probability of volunteering in the past month. Error bars represent 95% confidence intervals. All other model covariates are set at their means.

Figure 1 and Table S6 show the results of growth curves, adjusting only for the year of study entry and self versus proxy reporter. The intercepts showed that some activities were more common than others 13 years before death. For example, the average participant had a 92% predicted probability of visiting with family or friends, but only a 25% predicted probability of volunteering. All 5 forms of social participation declined significantly in the linear dimension from their levels 13 years before death. The squared term was also significant for visiting family and friends, organized activities, going out, and volunteering. The significant squared term means that declines in participation decelerated in the last 5 years of life for visiting friends and family and accelerated in the last 5 years of life for the other 3 forms of participation. Thus, the rate or pace of decline varied across the 5 forms of social participation. Going out for enjoyment showed the most decline. The average participant had an 80% predicted probability of going out for enjoyment 13 years before death, but only a 50% predicted probability of going out for enjoyment in the last year before death.

**FIGURE 2 |:**
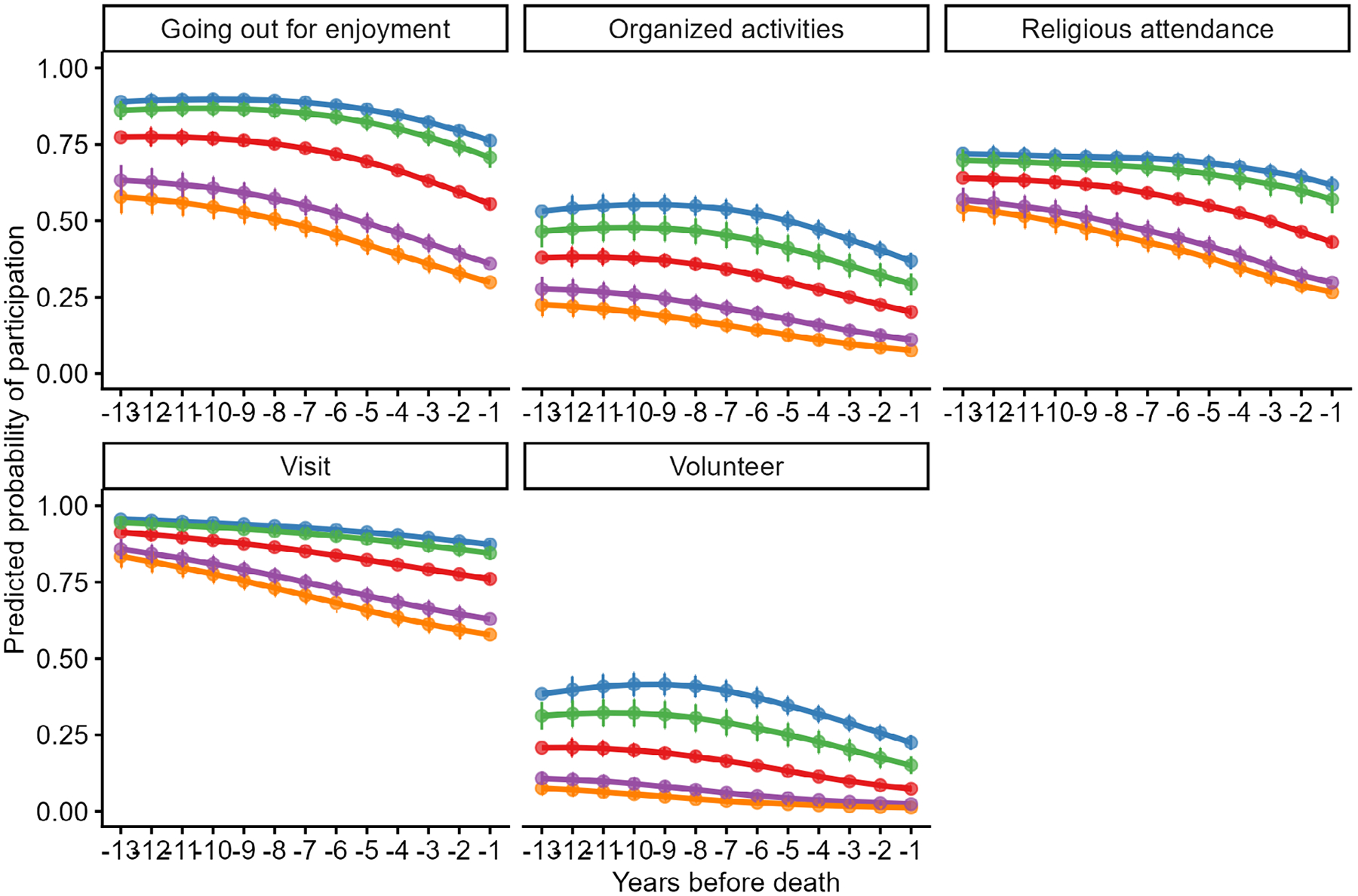
Predicted probabilities of participation in each social activity in the years before death, by physical and cognitive capacity. Red lines represent predicted probabilities for respondents with average physical and cognitive abilities throughout the study; blue lines represent predicted probabilities for respondents with high physical capacity and normal cognition throughout the study; green lines represent predicted probabilities for respondents with high physical capacity and probable dementia throughout the study; purple lines represent predicted probabilities for respondents with low physical capacity and normal cognition throughout the study; and orange lines represent predicted probabilities for respondents with low physical capacity and probable dementia throughout the study. Error bars represent 95% confidence intervals. All other model covariates are set at their means.

Figure 2 and Table S7 show the main effects of within- and between-person physical capacity and probable dementia on social participation, with adjustment for all covariates. Within-person improvement in physical capacity was significantly associated with a greater frequency of all 5 forms of social participation. For example, for the average person 2 years before death, an improvement in physical capacity from the mean (5) to a standard deviation above the mean (9) was associated with an increase in the predicted probability of attending religious services from 54% to 60%. Between-persons, individuals with better average physical capacity in the 5 years before death were most likely to participate in all 5 social activities. For example, for the average person 2 years before death, the predicted probability that a person with average physical capacity attended religious services was 44%, and the predicted probability that a person with physical capacity one standard deviation above average was 56%.

Within-person probable dementia (i.e., dementia onset) was statistically significantly associated with lower probabilities of participating in organized activities and of volunteering (*p* < 0.05). Both associations were too weak to be considered clinically significant, however. Between-person differences in probable dementia were significantly negatively associated with all 5 social activities. For example, for the average person 2 years before death, the predicted probability that a person with normal cognition throughout the study participated in organized activities was 25%, and the predicted probability that a person with probable dementia throughout the study participated in organized activities was 18%.

Moreover, the results show that when added together, variation in physical and cognitive function were associated with large differences in the predicted probability of participation. For example, a hypothetical person in the best possible health (i.e., an average physical capacity score of 12, normal cognition in all years) had a predicted probability of going out of 89% 13 years before death, and 76% the year before death. In comparison, a hypothetical person in the poorest possible health (i.e., an average physical capacity score of 0, probable dementia in all years) had a predicted probability of going out of 58% 13 years before death, and 30% the year before death. That is, the probability of going out was higher for a person in the best possible health the year before death than it was for a person in the poorest possible health 13 years before death. Notably, the entire range of health was present in the data: 150 decedents (3%) had the best possible health, and 82 (2%) decedents had the poorest possible health.

Table S8 shows whether and how within- and between-person physical capacity and probable dementia altered the rate of change in social participation over the years approaching death. Several associations were statistically significant at *p* < 0.05 but were too weak to be considered clinically significant.

## 3 | Discussion

Our first goal was to examine how older adults’ participation in social activities changes in the years prior to death. Our findings indicated that participation in all five social activities declined during this final period of the life course, with two notable patterns. First, some forms of social participation remained accessible to most people even very close to death, while other social activities were rare, even a decade or more before death, such that declines resulted in a very low probability of participation near death. For example, the models predicted that the average person would have at least monthly in-person visits with friends and family and trips out for enjoyment in the year before death. These are forms of participation that friends and family may readily facilitate, reducing barriers to older adults’ participation [36]. Less common activities included organized activities and volunteering, which are formal, structured activities where one might meet new people, learn new skills, be physically active, or do meaningful work: all aspects of participation known to enhance health and longevity [37]. Intervention efforts may focus on raising levels of participation in formal activities in midlife or earlier, to create patterns of engagement that are sustainable until death [38].

Second, we might hope for maintained participation until some terminal drop close to the time of death; in fact, declines in all 5 activities became apparent by 5 years before death. Moreover, physical and cognitive function were strongly associated with levels of participation up to 13 years before death. These findings echo other calls for communities to better support opportunities for older adults, particularly those with health limitations, to be socially engaged [28, 39, 40]. Neighborhood-based interventions to improve physical accessibility and dementia awareness may be important avenues for supporting social engagement, particularly among older adults living independently in their communities [41].

Our second goal was to examine whether physical and cognitive function altered rates of change in social activity participation. We found minimal evidence that physical or cognitive function affected trajectories of decline, alongside strong evidence that physical or cognitive function affected initial levels of participation. As a result, declines of similar magnitude still resulted in markedly different probabilities of social activity engagement in the years before death. For example, people with the highest levels of physical and cognitive function had a 76% predicted probability of going out for enjoyment at least once a month in the year before death, while people with the poorest physical and cognitive function had only a 30% predicted probability of the same. While prior research has emphasized the protective effect of social activity engagement for physical and cognitive trajectories [42–44], our findings suggest that physical or cognitive function do not meaningfully alter social trajectories during the final years of life. As death approaches, social activity trajectories may vary more significantly based on life course transitions, such as widowhood or a move to residential care [13, 45], or psychosocial factors, such as resilience or sense of purpose [46], than on physical and cognitive function.

Several limitations provide directions for future studies. We modeled physical and cognitive function as additive, because multiplicative models would have required complex four-way interaction terms. However, many older adults experience impairments in both physical and cognitive function in later life, and their effects may not be independent of one another [47]. Examining intersectional effects could shed further light on the health profiles at the greatest risk of end-of-life declines in social activity engagement. Additionally, NHATS does not identify the cause of death or collect subjective assessments of perceived time horizons. Future studies that collect this information could better assess how respondents’ perceptions or awareness of approaching end-of-life shape changes in patterns of social engagement.

In this nationally representative sample, U.S. older adults’ engagement in five social activities—visiting with family or friends, religious service attendance, organized group activities, going out for enjoyment, and volunteering—declined in the 13 years prior to death. Growth curve models showed that physical and cognitive function affected initial levels of participation, and that between-person differences in participation based on physical and cognitive function were sustained until death. Community-based interventions to improve physical accessibility and dementia awareness may be important avenues for supporting social activity engagement among people in the last stage of life, particularly among older adults living independently in their communities.

## Supplementary Material

supplemental

Additional supporting information can be found online in the Supporting Information section. Table S1: Number of annual self-and/or proxy-reports completed before death, *N* = 4667 participants in the national health and aging trends study, 2011–2024. Table S2: Covariates used in analysis. Table S3: Rates of participation in the analytic sample. Table S4: Descriptive statistics, time-invariant measures by cohort of study entry. Table S5: Descriptive statistics, time-varying measures by number of years before death. Table S6: Logistic growth curves of 5 forms of social participation on years until death. Table S7: Logistic growth curves of five forms of social participation, main effects of functional ability. Table S8: Logistic growth curves of 5 forms of social participation, functional ability by time.

## Figures and Tables

**TABLE 1 |:** Descriptive statistics, 22,993 observations from 4667 decedents in the National Health and Aging Trends Study, 2011–2024.

	Mean (SD) or %
Time-invariant measures (*N* = 4667 participants)	
Physical capacity: person-level average (0 poorest to 12 best)	5 (4)
Percentage of the study having probable dementia	35
Birth year (range: 1904–1956)	1931 (9)
Female	56
Race and ethnicity: White/non-Hispanic	69
Race and ethnicity: Black/non-Hispanic	22
Race and ethnicity: Other	3
Race and ethnicity: Hispanic	6
Educational attainment: Less than high school	31
Educational attainment: High school graduate	28
Educational attainment: Some college	19
Educational attainment: Associate’s degree or more education	22
Number of children	3 (2)
Income (U.S. dollars)	$39,000 ($85,000)
Time-varying measures (*N* = 22,993 observations)	
Physical capacity (0 poorest to 12 best)	6 (4)
Probable dementia	27
Visit family/friends	78
Religious services	52
Organized activities	29
Going out for enjoyment	62
Volunteering	16
Proxy reporter	15
Provided care in the past month	10
Worked for pay in the past month	6
Type of residence: Community	85
Type of residence: Residential care	15
Marital status: Unmarried	62
Marital status: Married	38

## Summary

- Key points
  - In a nationally representative sample, U.S. older adults’ engagement in 5 social activities—visiting with family or friends, religious service attendance, organized group activities, going out for enjoyment, and volunteering—declined in the 13 years prior to death.
  - In growth curve models, there was strong evidence that physical and cognitive function affected initial levels of participation, but minimal evidence that physical or cognitive function affected trajectories of decline.
  - Visits with family and friends and going out for enjoyment emerged as the most common social activities maintained in the years before death.
- Why Does This Paper Matter?
  - This paper matters because remaining socially active may be especially critical for quality of life as death approaches, as social engagement affords access to necessary support, health advice, sense of autonomy, and personal resources (e.g., coping, resilience) that shape end-of-life experiences. Interventions that support social activity engagement for older adults living with a wide range of functional capacities may be most effective in yielding these benefits.
