# Supplementary material for: Trajectories of Social Activity Engagement and Physical and Cognitive Function During the Last Years of Life
Source: J Am Geriatr Soc. Author manuscript; Available in PMC 2026 Jun 10. (PMC13251543; doi:10.1111/jgs.70410)
Supplement: supplemental [file NIHMS2181516-supplement-supplemental.pdf]

SUPPLEMENTAL MATERIAL

Table S1

Number of Annual Self- and/or Proxy-Reports Completed Before Death, N *= 4,667 Participants in the National Health and Aging Trends Study, 2011-2024*

Number of reports	*n*	%
13	125	3
12	100	2
11	146	3
10	201	4
9	267	6
8	286	6
7	311	7
6	409	9
5	372	8
4	404	9
3	481	10
2	746	16
1	819	18

Note. The average participant completed 5 reports (*SD* = 3).

Table S2

Covariates Used in Analysis

Measure	Description	Options
Time-Invariant		
Year of study entry		2011; 2015; 2022; 2023
Year of birth	Self-reported at study entry	Centered at 1904 in regression models
Sex	Self-reported at study entry	Male/female
Race and ethnicity	Self-reported at study entry	White non-Hispanic; Black non-Hispanic; Hispanic; other/multiple race/ethnicity
Educational attainment	Self-reported highest degree completed at study entry	Less than high school; high school diploma; some college; associate's or higher college degree
Number of children	Living children and stepchildren at study entry	Capped at 5
Income	Self-reported total income for self and spouse before taxes, asked in 2011, 2013, 2015, 2017, 2019, 2021, 2022, 2023 and averaged across those years	U.S. dollars, logged in regression models
Time-Varying		
Respondent type		Self/proxy report
Provided care in the past month	Besides as a job or volunteer work, did you provide care for or look after an adult or child who cannot care for themselves?	Yes/no
Worked in the past month	Work for pay or in a business you own?	Yes/no
Type of residence	Updated annually	Community/residential care
Marital status	Self-reported annually	Unmarried/married

Table S3

Rates of Participation in the Analytic Sample

Number of annual reports possible (Year of death – year of study entry)	Total *N*	*N* completing maximum number of possible reports	% complete participation
13	139	125	90
12	102	93	91
11	157	140	89
10	203	191	94
9	281	256	91
8	292	271	93
7	330	298	90
6	419	383	91
5	377	344	91
4	397	362	91
3	480	445	93
2	715	695	97
1	775	775	100
Total	4,677	4,378	94

Note. Stated otherwise, the average participant completed 98% of possible reports.

Table S4

Descriptive Statistics, Time-Invariant Measures by Cohort of Study Entry

	2011	2015	2022	2023
	Mean (*SD*) or %	Mean (*SD*) or %	Mean (*SD*) or %	Mean (*SD*) or %
Physical capacity: person-level average (0 *poorest* to 12 *best*)	6 (4)	6 (4)	5 (4)	5 (4)
Percentage of the study having probable dementia	35	34	28	43
Birth year	1929 (8)	1933 (8)	1940 (9)	1940 (9)
Female	58	54	47	48
Race and ethnicity: White/non-Hispanic	71	68	56	46
Race and ethnicity: Black/non-Hispanic	22	22	27	21
Race and ethnicity: Other	2	4	6	4
Race and ethnicity: Hispanic	5	6	11	29
Educational attainment: Less than high school	32	28	26	27
Educational attainment: High school graduate	27	31	27	22
Educational attainment: Some college	19	19	20	18
Educational attainment: Associate's degree or more	21	22	28	34
Number of children	3 (2)	3 (2)	3 (2)	2 (2)
Income (U.S. dollars)	39,000 (98,000)	37,000 (42,000)	45,000 (53,000)	39,000 (34,000)
N	3,104	1,204	239	120

Table S5

Descriptive Statistics, Time-Varying Measures by Number of Years Before Death

	13	12	11	10	9	8	7	6	5	4	3	2	1
	Mean (*SD*) or %	Mean (*SD*) or %	Mean (*SD*) or %	Mean (*SD*) or %	Mean (*SD*) or %	Mean (*SD*) or %	Mean (*SD*) or %	Mean (*SD*) or %	Mean (*SD*) or %	Mean (*SD*) or %	Mean (*SD*) or %	Mean (*SD*) or %	Mean (*SD*) or %
Physical capacity (0 *poorest* to 12 *best*)	9 (3)	9 (4)	9 (4)	9 (3)	8 (4)	8 (4)	8 (4)	7 (4)	7 (4)	7 (4)	6 (4)	5 (4)	5 (4)
Probable dementia	4	8	8	7	10	12	13	18	20	25	29	33	44
Visit family/friends	91	88	89	87	84	84	84	83	82	77	76	75	72
Religious services	70	64	65	63	64	63	61	60	56	51	48	46	41
Organized activities	40	45	45	40	40	38	36	34	31	30	27	25	21
Going out for enjoyment	82	77	79	77	76	74	72	71	70	62	60	56	47
Volunteering	31	31	29	29	26	25	22	20	17	16	13	11	8
Proxy reporter	3	3	3	4	4	5	6	7	8	11	15	19	30
Provided care in the past month	21	20	17	15	16	13	14	14	12	10	8	7	5
Worked for pay in the past month	17	17	17	13	11	11	8	7	7	6	5	4	3
Lives in residential care facility (vs. community)	4	4	6	7	7	7	8	10	12	14	17	19	22
Married (vs. unmarried)	55	49	47	44	44	42	42	40	39	37	35	34	33
Number of observations	139	239	395	596	874	1,162	1,484	1,898	2,267	2,637	3,090	3,746	4,466

Table S6

Logistic Growth Curves of Five Forms of Social Participation on Years Until Death

	Visit family/friends	Religious services	Organized activities	Going out for enjoyment	Volunteering
	Odds Ratio (95% CI)	Odds Ratio (95% CI)	Odds Ratio (95% CI)	Odds Ratio (95% CI)	Odds Ratio (95% CI)
Time					
Linear rate of change per year until death	0.89*** (0.84-0.95)	0.72*** (0.67-0.78)	0.72*** (0.67-0.77)	0.69*** (0.66-0.73)	0.49*** (0.44-0.55)
Squared rate of change	0.9891*** (0.9832-0.9951)	1.0027 (0.9954-1.0101)	1.0164*** (1.0106-1.0234)	1.0072* (1.0017-1.0129)	1.0398*** (1.0306-1.0490)
Fixed Intercept	3.72 (3.20-4.31)	0.45 (0.37-0.55)	0.07 (0.06-0.09)	0.89 (0.77-1.02)	0.00 (0.00-0.00)
Random Intercept[a]	2.61 (2.17-3.13)	11.91 (10.25-13.85)	7.38 (6.47-8.42)	4.40 (3.77-5.14)	15.67 (13.51-18.18)
Random Slope (linear)[a]	0.05 (0.03-0.07)	0.29 (0.24-0.35)	0.11 (0.08-0.13)	0.06 (0.05-0.08)	0.19 (0.15-0.24)
Slope-Intercept Correlation[a]	0.12 (0.05-0.19)	0.22 (0.06-0.39)	0.30 (0.19-0.41)	0.13 (0.05-0.22)	0.76 (0.53-0.99)
Wald χ^2; df	257.14; 6	490.70; 6	199.75; 6	1,073.94; 6	372.64; 6

Notes. CI = confidence interval. All models control for year of study entry (2011, 2015, 2022, 2023) and respondent type (self or proxy).

a Random components are not expressed as odds ratios.

*** $p < .001$, ** $p < .01$, * $p < .05$

Table S7

Logistic Growth Curves of Five Forms of Social Participation, Main Effects of Functional Ability

	Visit family/friends	Religious services	Organized activities	Going out for enjoyment	Volunteering
	Odds Ratio (95% CI)	Odds Ratio (95% CI)	Odds Ratio (95% CI)	Odds Ratio (95% CI)	Odds Ratio (95% CI)
Time					
Linear rate of change per year until death	0.95 (0.90-1.01)	0.75*** (0.69-0.81)	0.71*** (0.66-0.76)	0.77*** (0.73-0.82)	0.57*** (0.51-0.63)
Squared rate of change	0.9895** (0.9836-0.9954)	1.0021 (0.9946-1.0096)	1.0175*** (1.0116-1.0235)	1.0069* (1.0015-1.0123)	1.0325*** (1.0235-1.0416)
Functional Ability					
Physical capacity (within-person)	1.10*** (1.07-1.12)	1.15*** (1.12-1.18)	1.10*** (1.08-1.13)	1.15*** (1.12-1.17)	1.21*** (1.17-1.26)
Physical capacity (between-person)	1.17*** (1.14-1.19)	1.29*** (1.23-1.34)	1.25*** (1.21-1.28)	1.26*** (1.23-1.29)	1.47*** (1.40-1.54)
Probable dementia (within-person)	1.07 (0.91-1.26)	0.98 (0.79-1.21)	0.78* (0.64-0.95)	1.03 (0.87-1.21)	0.68* (0.50-0.93)
Probable dementia (between-person)	0.75** (0.63-0.89)	0.65* (0.46-0.90)	0.53*** (0.41-0.69)	0.65*** (0.53-0.80)	0.36*** (0.24-0.54)
Time-Invariant Covariates					
Entered 2015[a]	0.98 (0.85-1.14)	0.91 (0.70-1.20)	0.89 (0.72-1.10)	0.96 (0.82-1.14)	0.89 (0.65-1.20)
Entered 2022[a]	0.90 (0.60-1.35)	1.61 (0.81-3.23)	0.90 (0.48-1.68)	1.00 (0.62-1.58)	0.93 (0.35-2.47)

Table S7 *cont'd*	Visit family/friends	Religious services	Organized activities	Going out for enjoyment	Volunteering
	Odds Ratio (95% CI)	Odds Ratio (95% CI)	Odds Ratio (95% CI)	Odds Ratio (95% CI)	Odds Ratio (95% CI)
Entered 2023[a]	0.57<br>(0.29-1.11)	1.21<br>(0.37-3.96)	0.37<br>(0.11-1.24)	1.03<br>(0.46-2.31)	0.82<br>(0.14-4.85)
Year of birth	0.98***<br>(0.97-0.99)	0.97**<br>(0.96-0.99)	0.97***<br>(0.96-0.98)	0.9851**<br>(0.9756-0.9947)	0.98<br>(0.97-1.00)
Female	1.69***<br>(1.47-1.95)	3.25***<br>(2.48-4.26)	2.65***<br>(2.16-3.25)	1.66***<br>(1.41-1.94)	1.90***<br>(1.42-2.54)
Black/non-Hispanic[b]	0.65***<br>(0.56-0.76)	3.73***<br>(2.74-5.09)	0.81<br>(0.64-1.02)	0.41***<br>(0.35-0.49)	0.70*<br>(0.49-0.99)
Other[b]	0.52**<br>(0.36-0.76)	1.88<br>(0.89-3.97)	0.78<br>(0.44-1.42)	0.64<br>(0.41-1.01)	0.79<br>(0.33-1.92)
Hispanic[b]	0.53***<br>(0.41-0.68)	1.80*<br>(1.06-3.03)	0.62*<br>(0.40-0.97)	0.42***<br>(0.31-0.57)	0.33**<br>(0.16-0.69)
High school graduate[c]	1.26**<br>(1.07-1.48)	2.35***<br>(1.71-3.23)	2.08***<br>(1.62-2.67)	1.60***<br>(1.33-1.93)	1.98***<br>(1.36-2.89)
Some college[c]	1.27*<br>(1.05-1.52)	1.85**<br>(1.29-2.64)	2.92***<br>(2.22-3.84)	1.80***<br>(1.46-2.23)	2.54***<br>(1.69-3.81)
Associate's degree +[c]	1.69***<br>(1.40-2.05)	2.39***<br>(1.67-3.43)	7.74***<br>(5.85-10.24)	2.14***<br>(1.73-2.66)	8.12<br>(5.41-12.17)
Number of children	1.21***<br>(1.17-1.26)	1.30***<br>(1.20-1.40)	1.05<br>(0.99-1.11)	1.06**<br>(1.02-1.11)	1.05<br>(0.96-1.14)
Income (U.S. dollars)	1.09**<br>(1.03-1.16)	1.02<br>(0.92-1.15)	1.04<br>(0.95-1.14)	1.19***<br>(1.11-1.28)	0.92<br>(0.80-1.05)
Time-Varying Covariates					
Proxy reporter	1.36***<br>(1.17-1.59)	0.35***<br>(0.28-0.44)	1.19<br>(0.97-1.48)	0.62***<br>(0.53-0.74)	0.20***<br>(0.12-0.33)

Table S7 *cont'd*	Visit family/friends	Religious services	Organized activities	Going out for enjoyment	Volunteering
	Odds Ratio (95% CI)	Odds Ratio (95% CI)	Odds Ratio (95% CI)	Odds Ratio (95% CI)	Odds Ratio (95% CI)
Provided care	1.38***<br>(1.16-1.65)	1.14<br>(0.92-1.41)	1.08<br>(0.92-1.28)	1.04<br>(0.88-1.22)	1.29*<br>(1.05-1.59)
Worked for pay	1.48**<br>(1.16-1.89)	1.68**<br>(1.23-2.29)	1.42**<br>(1.12-1.79)	1.48**<br>(1.17-1.88)	1.65***<br>(1.25-2.18)
Lives in residential care facility (vs. community)	0.86*<br>(0.74-0.99)	4.01***<br>(13.21-5.01)	5.72***<br>(4.76-6.88)	0.50***<br>(0.43-0.59)	1.48**<br>(1.10-1.99)
Married (vs. unmarried)	1.02<br>(0.89-1.16)	0.94<br>(0.75-1.18)	0.93<br>(0.78-1.11)	1.15<br>(0.99-1.32)	0.86<br>(0.67-1.11)
Fixed Intercept	0.51<br>(0.26-1.01)	0.02<br>(0.00-0.06)	0.00<br>(0.00-0.01)	0.05<br>(0.02-0.10)	0.00<br>(0.00-0.00)
Random Intercept[d]	2.35<br>(1.94-2.84)	12.18<br>(10.50-14.12)	6.21<br>(5.38-7.17)	3.93<br>(3.38-4.56)	11.81<br>(10.01-13.93)
Random Slope (Linear)[d]	0.04<br>(0.03-0.06)	0.26<br>(0.21-0.32)	0.09<br>(0.07-0.11)	0.05<br>(0.04-0.07)	0.16<br>(0.12-0.21)
Slope-Intercept Correlation[d]	0.15<br>(0.08-0.22)	0.31<br>(0.15-0.48)	0.35<br>(0.24-0.46)	0.22<br>(0.14-0.30)	0.71<br>(0.49-0.94)
Wald χ^2*; df*	895.22; 24	850.84; 24	1014.28; 24	1,875.69; 24	779.10; 24

Notes. CI = confidence interval.

a Reference category is: entered 2011.

b Reference category is: White non-Hispanic.

c Reference category is: less than high school.

d Random components are not expressed as odds ratios.

*** $p < .001$, ** $p < .01$, * $p < .05$

Table S8

Logistic Growth Curves of Five Forms of Social Participation, Functional Ability by Time

	Visit family/friends	Religious services	Organized activities	Going out for enjoyment	Volunteering
	Odds Ratio (95% CI)	Odds Ratio (95% CI)	Odds Ratio (95% CI)	Odds Ratio (95% CI)	Odds Ratio (95% CI)
Time					
Linear rate of change per year until death	0.85* (0.75-0.98)	0.76** (0.63-0.92)	0.63*** (0.54-0.75)	0.76*** (0.67-0.87)	0.40*** (0.30-0.53)
Squared rate of change	0.9974 (0.9838-1.0112)	0.9891 (0.9700-1.0087)	1.0297*** (1.0138-1.0459)	1.0043 (0.9911-1.0176)	1.0642*** (1.0373-1.0919)
Functional Ability					
Physical capacity (within-person)	1.15*** (1.08-1.21)	1.15*** (1.07-1.24)	1.17*** (1.09-1.25)	1.14*** (1.08-1.20)	1.21*** (1.09-1.34)
Phys (within-person) * linear	1.02 (0.99-1.05)	1.00 (0.97-1.03)	1.01 (0.99-1.05)	0.99 (0.97-1.02)	1.02 (0.98-1.06)
Phys (within-person) * Squared	0.99 (0.99-1.00)	1.00 (0.99-1.00)	0.99 (0.99-1.00)	1.00 (0.99-1.00)	1.00 (0.99-1.00)
Physical capacity (between-person)	1.19*** (1.14-1.23)	1.31*** (1.24-1.39)	1.27*** (1.21-1.33)	1.30*** (1.25-1.36)	1.60*** (1.47-1.75)
Phys (between-person) * linear	1.01 (0.99-1.03)	1.00 (0.98-1.03)	1.01 (0.99-1.03)	1.02 (0.99-1.03)	1.04* (1.01-1.07)
Phys (between-person) * Squared	0.99 (0.99-1.00)	1.00 (0.99-1.00)	1.00 (0.99-1.00)	1.00 (1.00-1.00)	0.9970* (0.9944-0.9997)
Probable dementia (within-person)	1.23 (0.80-1.89)	0.70 (0.40-1.22)	0.57* (0.33-0.99)	0.48** (0.31-0.75)	0.65 (0.26-1.61)

Table S8 *cont'd*	Visit family/friends	Religious services	Organized activities	Going out for enjoyment	Volunteering
	Odds Ratio (95% CI)	Odds Ratio (95% CI)	Odds Ratio (95% CI)	Odds Ratio (95% CI)	Odds Ratio (95% CI)
Dem (within-person) * linear	1.12 (0.92-1.35)	0.88 (0.68-1.12)	0.92 (0.73-1.16)	0.77** (0.63-0.93)	0.96 (0.67-1.38)
Dem (within-person) * Squared	0.99 (0.97-1.01)	1.01 (0.99-1.04)	1.00 (0.98-1.02)	1.02 (1.00-1.03)	1.00 (0.97-1.04)
Probable dementia (between-person)	1.17 (0.83-1.65)	0.54* (0.38-1.05)	0.72 (0.45-1.15)	0.53** (0.37-0.76)	0.28** (0.11-0.68)
Dem (between-person) * linear	1.19* (1.02-1.40)	0.84 (0.67-1.04)	1.14 (0.94-1.39)	0.85* (0.72-0.99)	0.91 (0.65-1.27)
Dem (between-person) * Squared	0.99 (0.98-1.01)	1.03** (1.01-1.06)	0.99 (0.97-1.01)	1.02* (1.00-1.04)	1.01 (0.98-1.04)
Time-Invariant Covariates					
Entered 2015[a]	0.98 (0.85-1.13)	0.91 (0.70-1.20)	0.88 (0.72-1.09)	0.96 (0.82-1.14)	0.89 (0.65-1.21)
Entered 2022[a]	0.89 (0.60-1.34)	1.60 (0.80-3.21)	0.87 (0.47-1.63)	0.96 (0.60-1.54)	0.96 (0.35-2.60)
Entered 2023[a]	0.55 (0.28-1.07)	1.21 (0.37-3.97)	0.36 (0.11-1.18)	1.02 (0.45-2.30)	0.85 (0.14-5.24)
Year of birth	0.98*** (0.97-0.99)	0.97** (0.96-0.99)	0.97*** (0.96-0.98)	0.99** (0.98-0.99)	0.98 (0.97-1.00)
Female	1.69*** (1.47-1.95)	3.25*** (2.47-4.26)	2.64*** (2.15-3.24)	1.66*** (1.42-1.95)	1.90*** (1.42-2.54)
Black/non-Hispanic[b]	0.65*** (0.56-0.76)	3.73*** (2.73-5.09)	0.81 (0.64-1.02)	0.41*** (0.34-0.49)	0.69* (0.48-0.99)
Other[b]	0.53** (0.36-0.77)	1.87 (0.88-3.95)	0.78 (0.43-1.40)	0.63* (0.40-0.99)	0.78 (0.32-1.90)

Table S8 *cont'd*	Visit family/friends	Religious services	Organized activities	Going out for enjoyment	Volunteering
	Odds Ratio (95% CI)	Odds Ratio (95% CI)	Odds Ratio (95% CI)	Odds Ratio (95% CI)	Odds Ratio (95% CI)
Hispanic[b]	0.53*** (0.41-0.68)	1.80* (1.07-3.04)	0.63* (0.40-0.97)	0.42*** (0.30-0.57)	0.33** (0.16-0.68)
High school graduate[c]	1.25** (1.06-1.47)	2.36*** (1.72-3.25)	2.08*** (1.62-2.67)	1.61*** (1.33-1.94)	1.97*** (1.35-2.88)
Some college[c]	1.27* (1.05-1.52)	1.85** (1.30-2.65)	2.93*** (2.22-3.85)	1.82*** (1.47-2.25)	2.53*** (1.68-3.80)
Associate's degree +[c]	1.69*** (1.40-2.04)	2.41*** (1.68-3.45)	7.75*** (5.86-10.24)	2.16*** (1.74-2.69)	8.23*** (5.48-12.37)
Number of children	1.21*** (1.17-1.26)	1.30*** (1.21-1.40)	1.05 (0.99-1.11)	1.07** (1.02-1.11)	1.05 (0.97-1.14)
Income (U.S. dollars)	1.09** (1.03-1.16)	1.03 (0.92-1.15)	1.04 (0.95-1.15)	1.19*** (1.11-1.29)	0.91 (0.79-1.04)
Time-Varying Covariates					
Proxy reporter	1.29** (1.10-1.51)	0.36*** (0.28-0.45)	1.19 (0.96-1.48)	0.65*** (0.55-0.77)	0.21*** (0.12-0.34)
Provided care	1.38*** (1.15-1.64)	1.14 (0.92-1.41)	1.08 (0.92-1.28)	1.04 (0.89-1.23)	1.30* (1.05-1.60)
Worked for pay	1.46** (1.14-1.86)	1.69** (1.24-2.31)	1.41** (1.11-1.78)	1.49** (1.17-1.89)	1.66*** (1.26-2.20)
Lives in residential care facility (vs. community)	0.85* (0.74-0.99)	4.01*** (3.21-5.02)	5.71*** (4.74-6.87)	0.51*** (0.43-0.59)	1.52** (1.13-2.05)
Married (vs. unmarried)	1.01 (0.88-1.16)	0.94 (0.75-1.17)	0.92 (0.77-1.11)	1.14 (0.98-1.32)	0.87 (0.67-1.12)

Table S8 *cont'd*	Visit family/friends	Religious services	Organized activities	Going out for enjoyment	Volunteering
Fixed Intercept	0.40	0.02	0.00	0.04	0.00
	(0.20-0.82)	(0.00-0.06)	(0.00-0.01)	(0.02-0.10)	(0.00-0.00)
Random Intercept[d]	2.28	12.27	6.15	4.09	12.23
	(1.88-2.78)	(10.57-14.25)	(5.31-7.11)	(3.50-4.77)	(10.32-14.49)
Random Slope (Linear)[d]	0.04	0.26	0.09	0.05	0.16
	(0.03-0.06)	(0.21-0.32)	(0.07-0.12)	(0.04-0.07)	(0.12-0.21)
Slope-Intercept Correlation[d]	0.14	0.33	0.34	0.24	0.76
	(0.07-0.21)	(0.16-0.49)	(0.23-0.45)	(0.15-0.33)	(0.52-1.00)
Wald χ^2; *df*	892.61; 32	858.14; 32	1027.27; 32	1,874.76; 32	764.52; 32

Notes. CI = confidence interval.

a Reference category is: entered 2011.

b Reference category is: White non-Hispanic.

c Reference category is: less than high school.

d Random components are not expressed as odds ratios.

*** $p < .001$, ** $p < .01$, * $p < .05$
